# Supplementary material for: Dose blockchain-based agri-food supply chain guarantee the initial information authenticity? An evolutionary game perspective
Source: PLoS One. 2023 Jun 29;18(6):e0286886. doi: 10.1371/journal.pone.0286886 (PMC10310013; doi:10.1371/journal.pone.0286886)
Supplement: Attachment — Submitted filename: [0409]Response.(1).docx [file pone.0286886.s001.docx]

**Detailed Response to the Editors and Reviewers**

Dear Editors and Reviewers,

We greatly appreciated your constructive and critical remarks on the manuscript. The comments and suggestions have helped improve the contributions of the paper. Thank you sincerely.

Following your recommendations and suggestions, we have made an extensive revision of the manuscript. The paper is also thoroughly proofread to rectify any grammatical and typographical errors. We also provide detailed responses to the editors’ and reviewers’ comments. For easy identification, major revisions are marked in blue in the manuscript. Referee comments are in italics in this response. Quotes from the paper are in blue in this report. We hope the revised manuscript meets the publication standards for ***PLOS ONE***.

Thanks again for your recommendations.

Sincerely

Lindong Ma

Attachments

Response to Reviewer # 1……… 02

Response to Reviewer # 2……… 03

Response to Reviewer # 3……… 07

**Response to Reviewer 1**

**Reviewer 1:** *Congratulate you on the rigor of your reasoning. Your argumentative logic is particularly robust. The mathematical demonstration is of high quality. The different scenarios are plausible.However, you avoid an issue that I think is important to mention in the risk management section. You say that there is no transparency in the supply chain of the food industry... But you don't say why? Your article would benefit from stating the possible reasons for this situation. At this stage, I don't see what would be the reasons that could motivate actors to use the blobkchain unless they are forced to do so...Sometimes secrecy is the very condition of value creation.... What do you have to say about this?*

Response: We sincerely thank you for the comments and suggestions. The reason that why there is no transparency in the supply chain of the food industry has been added in introduction section.“Due to the complex structure of the AFSC and incomplete information systems, agricultural product supply information is discrete and difficult to share. ”The reason that could motivate actors to use the blobkchain has been also added in introduction section.“The blockchain can reduce the management and control of core enterprises in the agricultural product supply chain by using features such as decentralization and smart contracts, so that information can be shared in a timely manner, ensure the security and transparency of data, effectively solve the problem of information asymmetry in the agricultural product supply chain, eliminate information silo, improve the information synergy effect of each node, and improve the operation efficiency of the supply chain. ”

**Response to Reviewer 2**

**Reviewer 2:** *This study investigates the game theoretic setting in the agri-food supply chain field by analyzing the strategies of game players and their conditions,with the overall purpose to improve this supply chain network. Overall, the paperoffers interesting insights to understanding this issue.But some major and minor revisions are needed to improve the literature review,theoretical background of the approach, and modeling and simulation.*

Response: We sincerely thank you for the comments and suggestions. Revision based on your comments has helped us improve the manuscript significantly. We have revised the manuscript to address all of your concerns and suggestions.

**Reviewer Comment 2-1.** *In line 84, add the key contributions of your study.*

Response: We sincerely thank you for the comments and suggestions.The theoretical and practical implications of the study has been revised in introduction section. “The contributions of this article are as follows: (1) Theoretical aspects. This study focuses on the bilateral evolutionary game analysis of AFSC quality and safety management under blockchain technology, which enriches the literature on agricultural product quality and safety management from the perspective of blockchain technology application, and provides a new perspective and thinking for the research of AFSC information sharing. (2)Practical aspects. This study examines the factors that influence the initiative and enthusiasm of participants in the AFSC to share initial real information, providing some inspiration for the formulation of government regulatory strategies and the decision-making of enterprises in blockchain technology. It can better implement the application of blockchain technology in the AFSC, improve the overall efficiency of the AFSC, and promote the healthy development of agricultural products.”

**Reviewer Comment 2-2.** *The section 2.2 should highlight if your game is extensive or normal, and clarify who the public sector and who the private sector player is in the literature.The literature review is weak. About the blockchain technologies in agriculture,in the section 2.1 clarify on the sustainability and governance impacts. Also, start the section 2.2 saying that the games can be cooperative and non-cooperative and mention where your proposed model stands in. Also, highlight if your game is extensive or normal. Also, clarify who the public sector and who the private sector player is in the literature, since the game theory models much depend on this. There are a lot of studies on this issue in the public-private partnership literature, which can be briefly added into the section 2.2. The following studies should be added to improve these points:*

*- "Climate-smart agriculture using intelligent techniques, blockchain and Internet of*

*Things: Concepts, challenges, and opportunities" in TETT 2022 journal.*

*- "A review of the use of game theory in project management" in JME 2022 journal.*

*- "Analysis on the stability and evolutionary trend of the symbiosis system in the*

*supply chain of fresh agricultural products" in PO 2020 journal.*

*- "A meta-analysis of the public-private partnership literature reviews: exploring the*

*identity of the field" in IJSPM 2022 journal.*

Response: Thanks for your kindly help and for providing us with these useful reference papers. We have systematically sorted out and updated the literature.The section 2.1 has been revised in this paper.“Currently, academic research on the application of blockchain in the field of AFSCs focuses on application scenarios and effects. On one hand, representative perspectives on application scenarios include constructing a hierarchical structure model of “blockchain + AFSC”Ronaghi（Bals, L.,2019,Srai, J.S.,2018）[29,30], building a quality-and-safety-traceability system for agri-food( Yu, C. et al.,2020,Chu, Y,2019 )[31,32],constructing the traceability system of the fruit supply chain(Yang, X. et al,2021;Dhingra, V.2021)[33,34], and a blockchain-driven information-supervision model for the rice supply chain(Wang, J. et al,2022 )[35]. On the other hand, representative perspectives on the improvement of the AFSC operation by embedding blockchain technology include the industrial chain governance mechanism（Behzadi, G. et al,2017;Alzoubi, H.M.2019) [36,37],traceability solution(Sun, Y. et al,2018，Sun, B.et al,2020) [38],reducing the management costs and operational risks(Alkahtani, M,2021) [39], and setting pricing and ordering decisions(Niu, B. et al,2021)[40].”

The section 2.2 has been revised in this paper.“The research on the cooperation modes and game relationships among the participants in the AFSC includes : Van Ozkan-Canbolat, E. et al.(2016);Ketchen, D.J(2021) [41,42] built a multi-party evolutionary game model to determine whether the participants in the AFSC keep their promises and analyzed the influencing factors behind whether the participants in the AFSC follow the agreement to share information. Ho, K.L.P., et al.(2017), Wang, C. et al.(2017) [43,44] took the fresh agri-food supply chain as an example to build a game model and discuss decision-making behavior from the perspective of sharing preconditions and conditions, incentive mechanisms, channels, and platforms. The above studies either carried out game analysis from a static point of view or performed the dynamic analysis of information sharing among supply-chain nodes. Shahid, A. et al.(2020);Akın Ateş, M.(2021) [45,46] used a collaborative game model to study the stability of internal contractual cooperation in the supply chain of ecological agri-food and analyzed how blockchain technology can guarantee the stable operation of the ecological AFSC. Bergen, M.et al.(2019), and Wu, Y. et al.(2021) [47,48]built a game model of financial sharing behavior between agri-food suppliers and processors and analyzed the stability of the model to promote the smooth implementation of financial sharing in the AFSCs. Feixiao Wang,et al.(2022),and Zheng, Yi Xu,et al.(2023) built a evolutionary game to analyzed the stability of the model to promote quality of agricultural products in supply chain.

Above all,These studies covered most aspects of the application of blockchains in the field of agricultural supply chains but lacked systematic and dynamic research methods.and these studies did not analyze the feasibility of the implementation of blockchain in the AFSC. In China, the participants in the supply of agri-food, such as farmers, are scattered and small in scale. Since they lack capital, technology, information, and other aspects, it is difficult for them to integrate effectively into the blockchain [49,50]. what is more, there is no dynamic evolution analysis on the confidence sharing of the AFSC embedded in blockchain technology [51,52]. Additionally, there is no stability analysis on the sharing of the initial real information of each node enterprise in the AFSC in the blockchain.”

**Reviewer Comment 2-3.** *Is your model cooperative or non-cooperative? Please clarify this in the introduction to your model in the section 3.1*

Response: Many thanks for your advice here. We have discussed this in the section 3.1. “the relationship between nodes in the AFSC is reduced to a symbiotic relationship because of the close interdependence among them.......The two-game agents have bounded cooperative and rationality ”

**Reviewer Comment 2-4.** *Please clarify how the equilibrium can be reached in the matrix in Table 2 .*

Response: We sincerely thank you for the comments and suggestions. We have clarified this in Figure 1. Adecision tree demonstrating the players’ payoff.

**Reviewer Comment 2-5.** *In the section 3.3, define the concept of "stability", even if it leads to robust points to account for minor disturbances, but what is the stability in your study?*

Response: Thanks for your kindly suggestion here. In the paper,(1) $x=0 and x=1$ are two stable points of x.$(2) y=0 and y=1$ are two stable points of y.

**Reviewer Comment 2-6.** *In the section 4, it is not clear what are your input variables/data into the simulation. In the beginning of the section, in line 366-371 clarify this.*

Response: Many thanks for your advice here. We have clarified the source of the data in the beginning of the section.“In order to set the primitive parameters appropriately, the parameters of the model must meet economic hypotheses and empirical determinations.Based on the realistic implications of the model parameters and the historical data provided by Shaanxi Xinqiao Industrial Development Co., Ltd in xi’an, Shaanxi, China, the parameter values are initialized and assumed as follows:”

**Reviewer Comment 2-7.**  *In the conclusions section,The results are clear and the findings are interesting,and another future research can be to study downstream and upstream strategy players separately using such models as the Shapley or Rubinstein game models.*

Response: We sincerely thank you for the comments and suggestions. In the section 5.1,we have carefully revised“The results are clear and the findings are interesting, and This study provides a new perspective and idea for information sharing in the AFSC. It uses the evolutionary game method of both sides to conduct a dynamic and quantitative study on the strategy selection of participants in the AFSC under the background of blockchain technology, and analyzes the factors that affect the initiative and enthusiasm of participants in the AFSC to share initial real information, providing practical suggestions for improving the amount of information sharing and better promoting the quality and safety of agricultural products.”and we have another future research in the last paragraph of the article. “Lastly,another future research can be to study downstream and upstream strategy players separately using such models as the Shapley or Rubinstein game models.”

**Reviewer Comment 2-8.** *The English needs a proofreading by a native speaker.*

1. *Line 24 - remove the unnecessary full stop.*
2. *Line 25 - given a full name for the abbreviation.*
3. *The case is on Chine, so change the title to include "China" accordingly.*
4. *There can be other grammar issues to be fixed.*

Response: Many thanks for your advice here. We have asked professionals to carefully review and revise these grammar issues.And the title have been revised “Dose Blockchain-Based Agri-food Supply Chain Guarantee the Initial Information Authenticity in China? An Evolutionary Game Perspective.”

**Response to Reviewer 3**

**Reviewer Comment 3-1.** *The paper also has many writing errors and disfluencies, such as: Line24：“agri-food” should be capitalized as “Agri-food”; Line 146 “when” should becapitalized as“When”..*

Response: Many thanks for your advice here. We have asked professionals to carefully review and revise the article about grammar issues.

**Reviewer Comment 3-2.** *The literature is not comprehensive enough and limited in amount and without a summary of the latest research and to present research gaps, it is recommended to enrich the content of the literature review.*

Response: Thanks for your kindly help and for providing us with these useful reference papers. We have systematically sorted out and updated the literature. “RThe section 2.1 has been revised in this paper.“Currently, academic research on the application of blockchain in the field of AFSCs focuses on application scenarios and effects. On one hand, representative perspectives on application scenarios include constructing a hierarchical structure model of “blockchain + AFSC”Ronaghi（Bals, L.,2019,Srai, J.S.,2018）[29,30], building a quality-and-safety-traceability system for agri-food( Yu, C. et al.,2020,Chu, Y,2019 )[31,32],constructing the traceability system of the fruit supply chain(Yang, X. et al,2021;Dhingra, V.2021)[33,34], and a blockchain-driven information-supervision model for the rice supply chain(Wang, J. et al,2022 )[35]. On the other hand, representative perspectives on the improvement of the AFSC operation by embedding blockchain technology include the industrial chain governance mechanism（Behzadi, G. et al,2017;Alzoubi, H.M.2019) [36,37],traceability solution(Sun, Y. et al,2018，Sun, B.et al,2020) [38],reducing the management costs and operational risks(Alkahtani, M,2021) [39], and setting pricing and ordering decisions(Niu, B. et al,2021)[40].”

The section 2.2 has been revised in this paper.“The research on the cooperation modes and game relationships among the participants in the AFSC includes : Van Ozkan-Canbolat, E. et al.(2016);Ketchen, D.J(2021) [41,42] built a multi-party evolutionary game model to determine whether the participants in the AFSC keep their promises and analyzed the influencing factors behind whether the participants in the AFSC follow the agreement to share information. Ho, K.L.P., et al.(2017), Wang, C. et al.(2017) [43,44] took the fresh agri-food supply chain as an example to build a game model and discuss decision-making behavior from the perspective of sharing preconditions and conditions, incentive mechanisms, channels, and platforms. The above studies either carried out game analysis from a static point of view or performed the dynamic analysis of information sharing among supply-chain nodes. Shahid, A. et al.(2020);Akın Ateş, M.(2021) [45,46] used a collaborative game model to study the stability of internal contractual cooperation in the supply chain of ecological agri-food and analyzed how blockchain technology can guarantee the stable operation of the ecological AFSC. Bergen, M.et al.(2019), and Wu, Y. et al.(2021) [47,48]built a game model of financial sharing behavior between agri-food suppliers and processors and analyzed the stability of the model to promote the smooth implementation of financial sharing in the AFSCs. Feixiao Wang,et al.(2022),and Zheng, Yi Xu,et al.(2023) built a evolutionary game to analyzed the stability of the model to promote quality of agricultural products in supply chain.

Above all,These studies covered most aspects of the application of blockchains in the field of agricultural supply chains but lacked systematic and dynamic research methods.and these studies did not analyze the feasibility of the implementation of blockchain in the AFSC. In China, the participants in the supply of agri-food, such as farmers, are scattered and small in scale. Since they lack capital, technology, information, and other aspects, it is difficult for them to integrate effectively into the blockchain [49,50]. what is more, there is no dynamic evolution analysis on the confidence sharing of the AFSC embedded in blockchain technology [51,52]. Additionally, there is no stability analysis on the sharing of the initial real information of each node enterprise in the AFSC in the blockchain.”

**Reviewer Comment 3-3.***The variable definition in Table 1. does not match the case of the actual variable in the paper, for example, “X/Y ” should be changed to “x/y”, “M” should be “m”.*

Response: Thanks for your kindly suggestion here. This passage has been revised in Table 1.

**Reviewer Comment 3-4.** *Figure 1. missing "P" in the inequality sign of (c)..*

Response: We sincerely thank you for the comments and suggestions. PDF format conversion caused 'p' to be lost, now missing "P" has been added in the Figure 2(c) .

**Reviewer Comment 3-5.** *Figure 4. has two diagrams without separate labels.*

Response: Sincere thanks for your careful review. Both of the figures indicate that “evolution results under the initial parameter setting s”in different form.

**Reviewer Comment 3-6.** *Line 200 F(x) and Line 252 F(y) should be expressed as F_1_(x,y) and F_2_(x,y),respectively.*

Response: We sincerely thank you for the comments and suggestions. *F(x)* and *F(y)* have been revised at the corresponding position.

**Reviewer Comment 3-7.** *The overall volume of the paper is slightly less, and it is suggested to add more contents, such as using the three-way evolutionary game approach to study the game strategy selection problem of three groups.*

Response: Sincere thanks for your careful review. we have another future research in the last paragraph of the article. “ First, a project that extends the current model setting by incorporating government and logistics enterprises to construct a tripartite evolutionary game model is worth investigating.”

**Reviewer Comment 3-8.** *- The text could be reduced in sub-section 2.1There are many studies on evolutionary games that choose tripartite subjects, such as Zheng, Y et al. (2023) and Wang, F et al. (2022) which addresses blockchain traceability of agricultural supply chains and quality and safety of agricultural products, respectively..*

Response: Thanks for your kindly help and for providing us with these useful reference papers. We have carefully reviewed these literature and further improved the article accordingly.
